# Global Policy and Advocacy Initiatives for Improving Kidney Care: Report from the 2023 International Society of Nephrology Global Kidney Health Atlas

**DOI:** 10.34067/KID.0000000651

**Published:** 2024-11-19

**Authors:** Marina Wainstein, Sophanny Tiv, Silvia Arruebo, Fergus J. Caskey, Sandrine Damster, Jo-Ann Donner, Zaghloul Gouda, Vivekanand Jha, Adeera Levin, Masaomi Nangaku, Syed Saad, Feng Ye, Ikechi G. Okpechi, Aminu K. Bello, David W. Johnson, Valerie A. Luyckx

**Affiliations:** 1 Faculty of Medicine, University of Queensland, Brisbane, Queensland, Australia; 2 Division of Nephrology and Immunology, Faculty of Medicine and Dentistry, University of Alberta, Edmonton, Alberta, Canada; 3 The International Society of Nephrology, Brussels, Belgium; 4 Population Health Sciences, Bristol Medical School, University of Bristol, Bristol, United Kingdom; 5 Department of Nephrology, Damanhour Medical National Institute, General Organization of Teaching Hospitals and Institutes, Damanhour, Egypt; 6 George Institute for Global Health, University of New South Wales (UNSW), New Delhi, India; 7 School of Public Health, Imperial College, London, United Kingdom; 8 Manipal Academy of Higher Education, Manipal, India; 9 Division of Nephrology, Department of Medicine, University of British Columbia, Vancouver, British Columbia, Canada; 10 Division of Nephrology and Endocrinology, The University of Tokyo Graduate School of Medicine, Tokyo, Japan; 11 Department of Kidney and Transplant Services, Princess Alexandra Hospital, Brisbane, Queensland, Australia; 12 Centre for Kidney Disease Research, University of Queensland at Princess Alexandra Hospital, Brisbane, Queensland, Australia; 13 Translational Research Institute, Brisbane, Queensland, Australia; 14 Australasian Kidney Trials Network at the University of Queensland, Brisbane, Queensland, Australia; 15 University Children's Hospital, University of Zurich, Zurich, Switzerland; 16 Department of Public and Global Health, Epidemiology, Biostatistics and Prevention Institute, University of Zurich, Zurich, Switzerland; 17 Renal Division, Brigham and Women's Hospital, Harvard Medical School, Boston, Massachusetts; 18 Department of Paediatrics and Child Health, University of Cape Town, Cape Town, South Africa

**Keywords:** AKI, CKD, health policy, kidney failure

## Abstract

**Key Points:**

- Inclusion and prioritization of CKD and kidney failure within national health strategies are generally lacking.
- Countries with CKD-specific strategies tend to include and fund a broader spectrum of kidney disease populations and kidney care.
- Greater global and national prioritization of kidney health are required to reduce global inequities in access to kidney care.

**Background:**

National strategies to address CKD are crucial to support kidney health. Lack of political support in the form of policy decisions and funding leads to fragmentation of kidney care and catastrophic health expenditure. This study used data from the third iteration of the International Society of Nephrology Global Kidney Health Atlas to obtain a global overview of the existence and reach of national strategies for kidney care.

**Methods:**

We leveraged data from an international survey of stakeholders (clinicians, policymakers, and patient advocates) conducted by the International Society of Nephrology between July and September 2022. Data were extracted on existence and scope of national noncommunicable disease (NCD) and/or CKD-specific strategies and policies, as well as recognition of kidney disease as a national health priority through participant perception and existence of CKD advocacy groups.

**Results:**

Overall, stakeholders from 167 countries responded to the survey, representing 97.4% of the global population. National strategies for NCDs were reported by 56% of countries. In 29% of countries, CKD was addressed within an NCD strategy, whereas 25% of countries reported CKD-specific strategies. Countries with CKD-specific strategies were more likely to address all CKD populations (non–dialysis-dependent CKD, chronic dialysis, and kidney transplantation) compared with those with NCD strategies only (51.2% versus 19%). Of the 54% of countries with any CKD strategy, 89% reported public funding of the full spectrum of CKD care compared with 64% of those with no CKD strategy. Kidney failure, CKD, and AKI were reported to be recognized as national health priorities by 63%, 48%, and 19% of countries, respectively.

**Conclusions:**

The inclusion of CKD and kidney failure within national health strategies is frequently lacking. Countries with CKD-specific policies tend to include a broader spectrum of kidney disease populations and to fund kidney care more than those with CKD policies integrated within NCD strategies. Greater global and national prioritization of kidney health are required to reduce global inequities in access to kidney care.

## Visual Abstract

**Figure:**
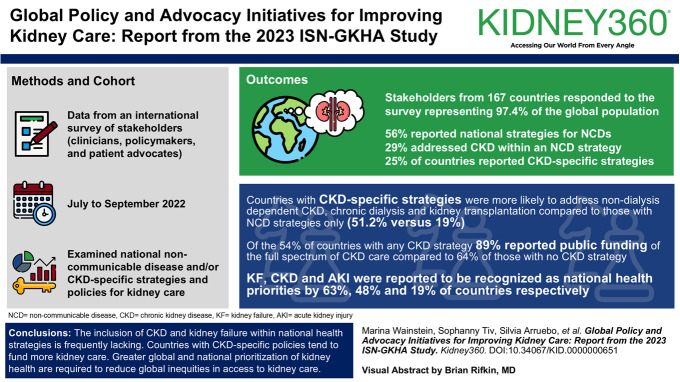

## Introduction

Kidney disease affects one in eight to ten people worldwide.^1,2^ Despite this, kidney disease has not been prioritized in the global noncommunicable diseases (NCDs) agenda, in part, because of lack of robust data on the disease burden and cost-effectiveness of treatment options, as well as a general tendency of communities and health systems to consider kidney disease predominantly in terms of dialysis and transplantation.^3^

According to the Global Burden of Disease study, around 3.6 million deaths in 2021 were attributed to kidney disease, which ranked as the eighth leading global risk factor of death.^4^ A diagnosis of kidney disease requires access to and use of diagnostic tests that are not available everywhere, and therefore, it is likely that many deaths from kidney disease remain uncounted.^5^ Indeed, extrapolations on the basis of expected incidence and prevalence of acute and chronic kidney failure (KF) estimate that around 1.7 million people per year may die of AKI and between 2 and 7 million may die of lack of access to KRT for KF each year.^3,6,7^ Most of these missed deaths occur in lower resource settings, which reflects the known inequitable access to KRT for KF, as well as global disparities in prevention, early detection, and appropriate treatment of CKD or its major risk factors, such as hypertension and diabetes.^8,9^ These facts, coupled with poor awareness among first-line clinicians and a nihilistic view that there is no treatment for kidney disease, keeps CKD in the shadows of many other NCDs. Accordingly, CKD is one of few conditions where the global age-standardized mortality rate has not declined, and kidney diseases are the leading causes of catastrophic health expenditure in low- and middle-income countries.^2,10^ Understanding the policy landscape related to CKD is necessary to develop advocacy strategies to improve awareness of kidney disease and improve access to and quality of kidney care.

The International Society of Nephrology-Global Kidney Health Atlas (ISN-GKHA) has completed three rounds of surveys to assess the global policy landscape regarding kidney health and kidney diseases. In the first iteration of the ISN-GKHA in 2016, among 116 responding countries, 36% identified CKD as a health priority.^11^ NCD strategies were in place in 59% of countries, with 44%, 55%, and 47% of countries reporting existence of a national strategy for improving the care of people with nondialysis CKD, dialysis, and kidney transplantation, respectively, and 49% having a strategy to improve identification of AKI. In the second ISN-GKHA iteration in 2018, among 154 responding countries, 51% identified CKD as a national priority.^12^ NCD strategies were reported in 47% of countries, whereas 34% reported CKD-specific policies. Overall, NCD and kidney-specific strategies and policies were more prevalent in higher resource settings.

The third iteration of the ISN-GKHA was completed soon after the end of the coronavirus disease 2019 pandemic in 2022.^8^ In this study, we report the findings of the third ISN-GKHA survey with regard to the presence and scope of NCD and CKD strategies, the relationship to public funding of kidney care, perceived recognition of kidney disease as a national health priority, and the existence of advocacy efforts within countries and regions.

## Methods

### Survey

Detailed methods of the ISN-GKHA have been published elsewhere.^13^ The ISN-GKHA data are derived from a multinational, cross-sectional online survey of opinion leaders in nephrology. Three key stakeholders, including a nephrology society leader, a policymaker, and a leader of a patient representative organization, were identified from 191 countries with International Society of Nephrology (ISN) affiliate societies that were invited to participate in the survey. The stakeholders were sent an electronic link to the survey's online portal through research electronic data capture (https://www.redcapcloud.com). The survey was available in English, French, and Spanish. The survey was conducted from July 1 to September 30, 2022, and was coordinated through ISN's ten regional boards (Africa, Eastern and Central Europe, Latin America, Middle East, North America and the Caribbean, North and East Asia, Oceania and South East Asia, Newly Independent States and Russia, South Asia, and Western Europe). Survey participants were asked to report whether NCDs, CKD, AKI, and KF were specifically addressed in their national health strategies and policies and the extent to which various kidney disease populations were included. Definitions of kidney care policies and strategies, as well as the different kidney disease populations used in the survey, are outlined in Supplemental Table 1. In addition, respondents were asked whether they felt CKD was prioritized in their country and whether advocacy initiatives were in place. Survey questions relating to the national policy landscape are outlined in Supplemental Table 2 and signposted throughout the Results section.

### Data Handling and Analysis

Responses to the French and Spanish surveys were translated into English by certified translators. Data from all individual surveys were then extracted into Microsoft Excel and checked for inconsistencies, missing data and duplicates before combining it into a single file to create the global database for analysis using Stata 17 software (Stata Corporation, 2017). Because each country was the unit of analysis, data from multiple respondents within the same country were merged into a single response after regional board representatives clarified any inconsistencies. Responses were summarized as counts and percentages of countries for each response. The results were then stratified by ISN region and World Bank country income group: low income, lower-middle income, upper-middle income, and high income (estimated in June 2022). Global estimates, regional estimates, and country income-level estimates are presented as medians and interquartile ranges. Survey results were not comparable across the three ISN-GKHA iterations because a different number of countries participated in each.

Given the variability in numbers of responses across regions, and in some cases, very small numbers, the data are presented descriptively. Statistical analyses were not performed to avoid overinterpretation of the data. To provide an intuitive representation of the global variability, data are presented as world maps with country responses indicated with shading or icons to illustrate distribution of policy strategies and of CKD populations covered.

### Ethics Approval and Consent to Participate

The University of Alberta Research Ethics Committee approved this project (protocol number: PRO00063121). Consent was not required by survey respondents. Our study did not report experiments on humans and/or the use of human tissue samples.

### Consent for Publication

No individual person's data were used. Consent was not required by survey respondents.

## Results

Responses were obtained from 167 countries, comprising 20 low-income (12%), 45 lower-middle–income (27%), 39 upper-middle–income (23%), and 63 high-income (38%) countries, representing 97.4% of the world's population. Of the 325 survey respondents, 273 were nephrologists (84%), followed by 18 non-nephrologist physicians (6%), six nonphysician health care professionals (2%), 14 administrators/policymakers (4%), and 16 others affiliated with advocacy groups for people with kidney disease (5%).

### Strategies and Policies

#### National NCD and CKD-Specific Strategies

**Figure 1:**
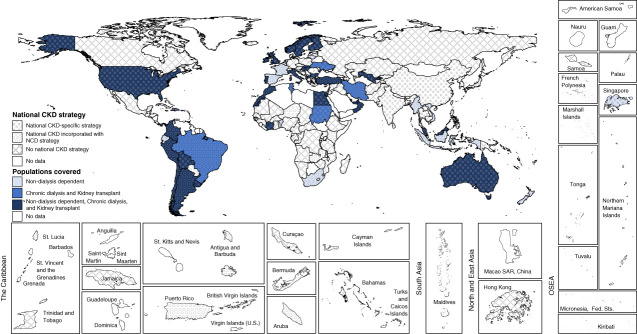
**Type of national CKD strategy and population covered.** NCD, noncommunicable disease.

Over half of all countries (56%, *n*=91) reported having a national strategy for NCDs in place, and a further 12% (*n*=19) had one under development (Supplemental Table 3). CKD-specific national strategies were reported by 25% countries (*n*=41), whereas 29% (*n*=47) reported CKD being incorporated within an NCD strategy (Table 1). The proportion of countries reporting any national CKD strategy declined with country income category (Table 1). In general, coverage of all CKD populations (CKD stages 1–5: non–dialysis-dependent CKD, chronic dialysis, and kidney transplantation) was more frequent in countries with CKD-specific strategies compared with countries where CKD was incorporated into an NCD strategy (51%, *n*=21 versus 19%, *n*=9, respectively; Figure 1 and Table 2). Nondialysis CKD was covered in 32% of countries where CKD was included within NCD strategies.

**Table 1:** Existence of a national strategy for improving the care of people with CKD

Regions and Populations Covered by Survey Responses	No	Yes, a National CKD-Specific Strategy Exists	Yes, but the CKD Strategy is Incorporated into an NCD Strategy that Includes Other Diseases	Unknown	Total
Overall	61 (38)	41 (25)	47 (29)	13 (8)	162
**ISN region**					
Africa	22 (56)	5 (13)	11 (28)	1 (3)	39
Eastern and Central Europe	4 (25)	6 (38)	5 (31)	1 (6)	16
Latin America	4 (19)	6 (29)	8 (38)	3 (14)	21
Middle East	4 (36)	4 (36)	1 (9)	2 (18)	11
NIS and Russia	3 (30)	2 (20)	4 (40)	1 (10)	10
North America and the Caribbean	3 (25)	3 (25)	4 (33)	2 (17)	12
North and East Asia	1 (17)	3 (50)	1 (17)	1 (17)	6
OSEA	7 (39)	5 (28)	6 (33)	0	18
South Asia	3 (43)	2 (29)	2 (29)	0	7
Western Europe	10 (45)	5 (23)	5 (23)	2 (9)	22
**World Bank groups**					
Low income	12 (67)	2 (11)	4 (22)	0	18
Lower-middle income	20 (45)	10 (23)	12 (27)	2 (5)	44
Upper-middle income	11 (30)	8 (22)	13 (35)	5 (14)	37
High income	18 (29)	21 (33)	18 (29)	6 (10)	63

ISN, International Society of Nephrology; NCD, noncommunicable disease; NIS, Newly Independent States; OSEA, Oceania and South East Asia.

**Table 2:** Populations covered by CKD strategy type

Populations Covered in Each Strategy	Type of Strategy^a^			
	CKD-Specific (*n*=41)		NCD (*n*=47)	
	*n*	%	*n*	%
Nondialysis	4	10	15	32
Nondialysis and dialysis	3	7	2	4
Nondialysis and transplant	0	0	0	0
Nondialysis, dialysis, and transplant	21	51	9	19
Dialysis	0	0	2	4
Dialysis and transplant	5	12	2	4
Transplant	0	0	2	4
None	8	20	15	32

NCD, noncommunicable disease.

a Each country is counted once for each type of CKD strategy based on the category selected.

#### CKD Strategies and Public Funding

**Figure 2:**
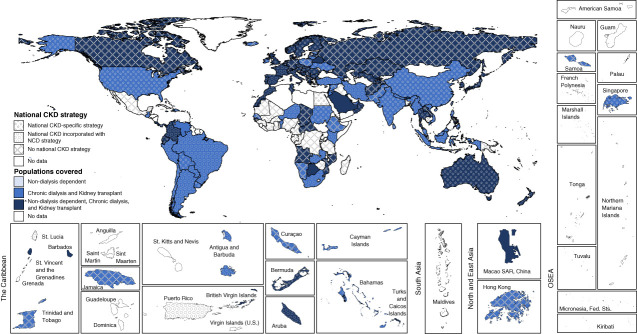
Association between existence and type of CKD strategy and public funding of CKD care.

Public funding for all forms of CKD care (KRT and nondialysis care) was reported by 89% (*n*=78/88) of countries with any national strategy for CKD compared with 64% (*n*=39/61) of countries without any type of CKD strategy. When stratified by strategy type, public funding for all forms of CKD care tended to be more common among countries with a CKD-specific strategy (49%, *n*=20) compared with those with CKD within a general NCD strategy (38%, *n*=18; Table 3), although proportions of countries funding KRT alone was similar (44% and 47%, respectively). Trends were, however, inconsistent within and between regions (Figure 2). With either type of CKD strategy, public funding of all forms of CKD care was less common among low-income and lower-middle–income countries.

**Table 3:** Populations funded publicly by CKD strategy type

Populations Funded Publicly	Type of Strategy^a^			
	CKD-Specific (*n*=41)		NCD (*n*=47)	
	*n*	%	*n*	%
Nondialysis	0	0	0	0
Nondialysis and dialysis	2	5	1	2
Nondialysis and transplant	0	0	0	0
Nondialysis, dialysis, and transplant	20	49	18	38
Dialysis	1	2	3	6
Dialysis and transplant	18	44	22	47
Transplant	0	0	0	0
None	0	0	3	6

NCD, noncommunicable disease.

a Each country is counted once for each type of CKD strategy based on the category selected.

#### National CKD Policies

CKD-specific policies (as opposed to strategies, defined in Supplemental Table 1) were reported by 37% (*n*=60) of countries, predominantly upper-middle–income and high-income countries. They were most prevalent in Eastern and Central Europe (63%, *n*=10), Newly Independent States and Russia (60%, *n*=6), and North and East Asia (67%, *n*=67). Only two low-income and 11 lower-middle–income countries reported CKD-specific policies. Policies were more commonly national compared with regional in reach (82% versus 22%, Supplemental Figure 1).

### Prioritization of Kidney Health and Advocacy Activity

**Figure 3:**
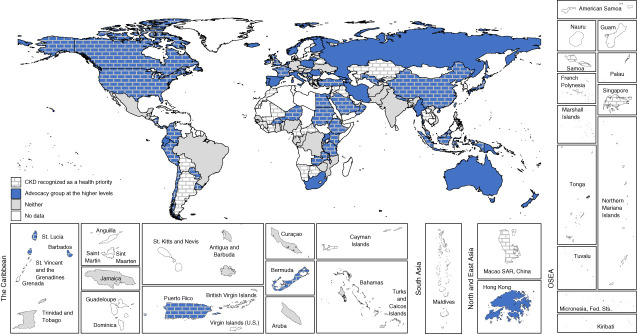
Association between national recognition of CKD and existence of advocacy groups.

**Figure 4:**
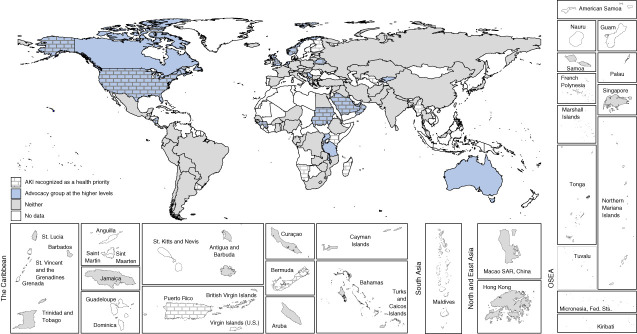
Association between national recognition of AKI and existence of advocacy groups.

**Figure 5:**
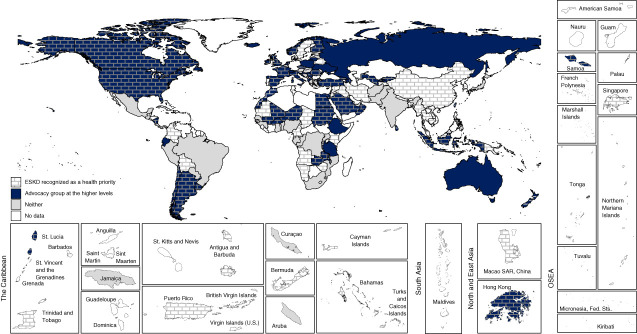
**Association between national recognition of KF and existence of advocacy groups.** KF, kidney failure.

Respondents from almost half of all participating countries (48%, *n*=78) answered that CKD was recognized as a health priority by their government, with the highest proportions in North and East Asia (83%, *n*=5) and the Middle East (82%, *n*=9). There was no discernible pattern between country income groups. The presence of an advocacy group for CKD prevention and care at the higher level of government (*e.g*., a parliamentary committee or a non-governmental organization was reported by 64 countries [40%]) (Figure 3). Less than one in five countries (19%, *n*=30) reported AKI being recognized as a national health priority, with only 11% (*n*=18) reporting the existence of an advocacy group to raise its profile (Figure 4). KF was recognized as a national health priority by 63% (*n*=102) of countries, with 34% (*n*=55) reporting activity of an advocacy group at the higher levels of government (Figure 5). Among countries that did not recognize kidney disease as a national health priority, an advocacy group at the higher level of government existed in only 27% (*n*=23) for CKD, 7% (*n*=9) for AKI, and 18% (*n*=11) for KF.

## Discussion

Despite kidney disease being an important public health concern with rising global mortality and catastrophic health expenditure for individuals needing to access kidney care, CKD is being overlooked in around half of countries globally. Only 29% of countries reported inclusion of CKD within their NCD strategy, and 25% of countries reported having a specific CKD strategy. Not surprisingly, these proportions varied by up to five- to ten-fold between individual LICs compared with HICs (data not shown).

Although there was some heterogeneity in the data, countries with a CKD strategy of any form (*i.e*., CKD-specific strategy or incorporated within an NCD strategy) were more likely to publicly fund some form of kidney care compared with those without a CKD strategy in place. In addition, countries with a national CKD-specific strategy were more likely to cover all CKD populations and publicly fund the full spectrum of care compared with those where CKD was incorporated into an NCD strategy. In countries where CKD was integrated into NCD strategies, however, nondialysis CKD care was covered in one third, which does demonstrate some awareness of the need to integrate CKD into NCD care (Table 2). Funding for KRT alone was reported by 44%–47% of countries with any CKD strategy (Table 3); this may suggest that, given the significant out-of-pocket costs required, countries may tend initially to prioritize funding of KRT over prevention and non-KRT care. This is a challenging circumstance, especially when budgets are limited, because funding for prevention and early CKD care would lead to a reduction in the downstream KF burden (and ultimately costs).^14^ Funding for earlier CKD care is indeed known to be cost-effective in multiple health systems.^15–17^ At the health systems level, integration of CKD into NCD programs could address CKD risk factors upstream, in primary care, which would minimize the risk of development and progression of CKD and mitigate the downstream cascade of complications.

CKD is preventable through public health measures and addressing many social determinants of health, it is easily detected with basic diagnostics, and it is treatable with generic medications.^18^ Care for people living with CKD must be integrated within the health system because CKD overlaps with many conditions, and care pathways should be streamlined to maximize efficiency, quality, and acceptability.^19,20^ Also importantly, a Health in All Policies approach is required for NCDs as a whole, but especially so for kidney disease, given that policies relating to aging, maternal and child health, commercial determinants of health, humanitarian emergencies, safe cities, affordable food and housing, education, and gender equity all affect kidney health.^3^

Having a national strategy for NCDs is an important first step toward addressing CKD and its risk factors, such as diabetes and hypertension, but this is not enough. Ideally, CKD policies should be comprehensively incorporated into overarching NCD strategies to capture and address risk factors, such as hypertension, diabetes, and obesity, early. However, given the budget implications and complexities inherent to the spectrum of kidney care, as well as additional risk factors which may be more common in lower resource settings, such as AKI, preeclampsia, and genetic predispositions, kidney-specific policies may have additional value.

**Figure 6:**
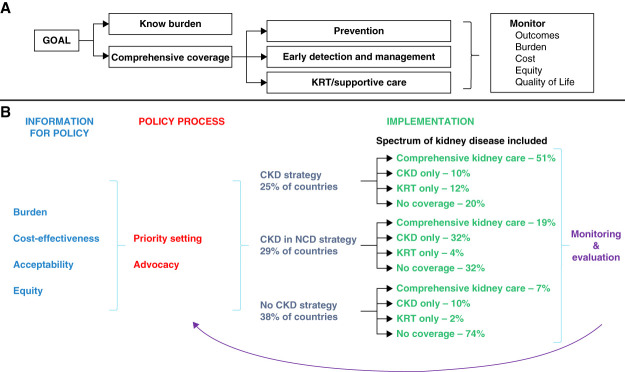
Summary figure of the policy implementation process to support kidney care programs.

Why some countries have NCD and CKD strategies and policies and others do not is not possible to determine from this study. The trends observed here are consistent with an analysis of NCD policies across Organization for Economic Co-operation and Development countries, where CKD was included within NCD policies of only three of 31 member states, compared with policies for cancer, cardiovascular disease and diabetes, and mental health and respiratory disease, being present in 25, 23, and 19 states, respectively,^19^ reflecting global prioritization of these conditions. The letter “K” is conspicuously missing from the alphabetical list of World Health Organization disease fact sheets, highlighting that kidney disease has been omitted from the global health agenda.^21^ Global prioritization drives country-level prioritization of diseases. In addition, policies may not be in place because local data regarding disease burden, cost-effectiveness and acceptability of therapies, and inequities may not be available (Figure 6). Such data are required to support fair and transparent priority setting and resource allocation and to inform kidney care program design, which must be monitored to determine its effect and effectiveness. It is clear that many gaps persist along this cycle, which the global kidney community must highlight and collaborate to address.

Country income category also seems to determine the existence of CKD strategies and policies, which declined with country income (Table 1). Kidney care is expensive; therefore, many countries have had to focus priorities elsewhere.^22–24^ Innovation and global collaboration are required to develop sustainable financing strategies to ensure equitable and affordable access to medications to slow progression of CKD, to specialist nephrology care, and to KRT for those who develop KF.^14,25,26^

Surveys such as the ISN-GKHA have many strengths and limitations. The strengths of this study include its comprehensive coverage of the global population across all regions and country income levels, the use of a validated survey, and the gathering of responses from multiple sources to characterize a country's profile in terms of kidney disease prioritization. The main limitations include the risk of response bias, both in terms of social desirability and the tendency to overestimate a country's public health image, and of knowledge bias, because many clinicians and health professionals may be unaware of the specific kidney disease policies and strategies in their own countries. The response rate from policymakers was low. While the survey provided definitions for strategies versus policies, we could not rule out the possibility of coding bias due to confusion between these terms. No statistical methods were used to evaluate comparisons given the small numbers of countries in each question and potential for overinterpretation.

The aim of this study was to investigate potential trends between existence of CKD strategies, the spectrum of kidney disease populations covered, and the availability of public funding for kidney care. In-depth characterization of a country's or region's existing CKD policy landscape would require rigorous triangulation with published literature and policy documents to validate survey responses, which was beyond the scope of the study. Furthermore, while we found an association between the level of public funding and type of national CKD strategy, we were unable to determine the direction of this association, such that it is difficult to know which came first—that is, whether policy development grew out of a need to contain existing costs or whether policy became the main driver for resource and service allocation. Despite these limitations, the ISN-GKHA is currently the only initiative to have systematically assessed the global status of kidney care and, therefore, represents the best current available data on this topic and generates many questions that require further study.

This study highlights the urgent need to support the policy-implementation cycle for kidney health and kidney care. Although acknowledging kidney disease within a national NCD strategy is an important and valuable step forward, this may not be enough to cover the full spectrum of risk factors of kidney disease and the complexities of KF care. Kidney-specific strategies and policies support funding and delivery of lifesaving kidney care. Prioritization of kidney disease at the global and government levels is urgently needed to improve equity in access to quality and affordable kidney care everywhere.

## Data Availability

Partial restrictions to the data and/or materials apply. The datasets used and analyzed during the current survey are available from the corresponding author on reasonable request.

## Supplemental Material

Supplemental Table 1. Survey question terminology definitions.

Supplemental Table 2. Survey questions.

Supplemental Table 3. Existence of a national strategy for noncommunicable diseases in 2022.

Supplemental Figure 1. Availability of CKD-specific policies.
